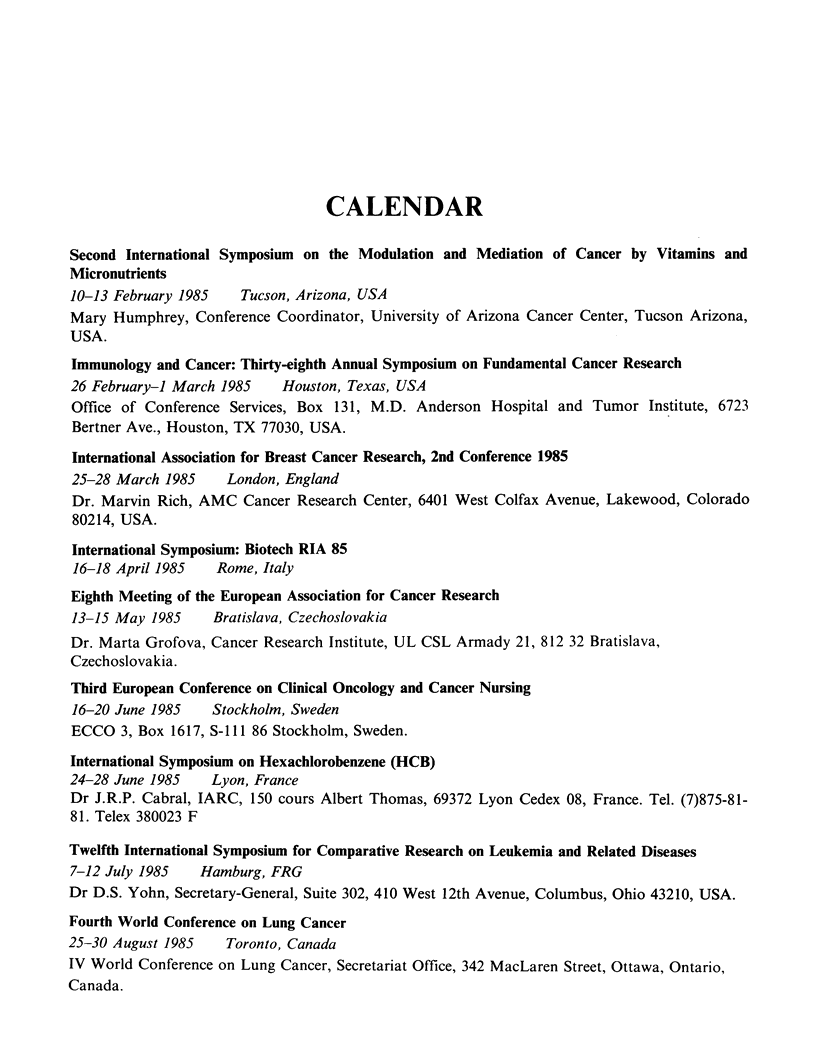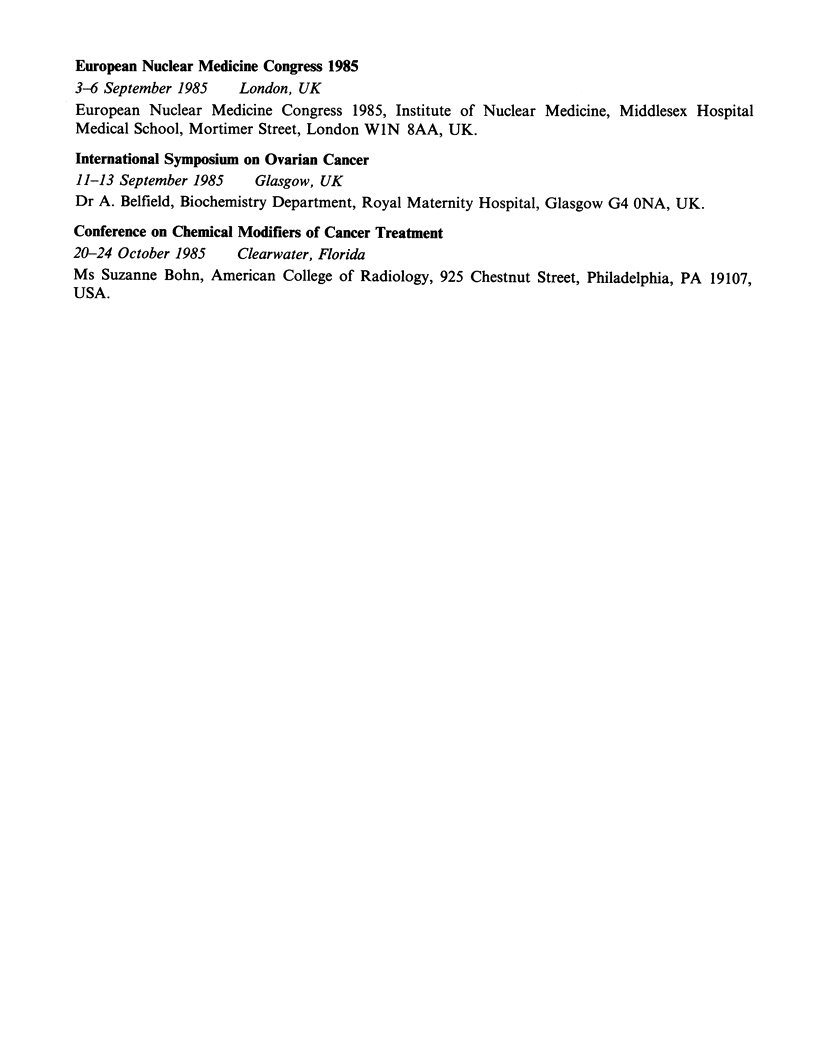# Calendar

**Published:** 1984-12

**Authors:** 


### CALENDAR

**Second International Symposium on the Modulation and Mediation of Cancer by Vitamins and Micronutrients**

*10–13 February 1985 Tucson, Arizona, USA*

Mary Humphrey, Conference Coordinator, University of Arizona Cancer Center, Tucson Arizona, USA.

**Immunology and Cancer: Thirty-eighth Annual Symposium on Fundamental Cancer Research**

*26 February–1 March 1985 Houston, Texas, USA*

Office of Conference Services, Box 131, M.D. Anderson Hospital and Tumor Institute, 6723 Bertner Ave., Houston, TX 77030, USA.

**International Association for Breast Cancer Research, 2nd Conference 1985**

*25–28 March 1985 London, England*

Dr. Marvin Rich, AMC Cancer Research Center, 6401 West Colfax Avenue, Lakewood, Colorado 80214, USA.

**International Symposium: Biotech RIA 85**

*16–18 April 1985 Rome, Italy*

**Eighth Meeting of the European Association for Cancer Research**

*13–15 May 1985 Bratislava, Czechoslovakia*

Dr. Marta Grofova, Cancer Research Institute, UL CSL Armady 21, 812 32 Bratislava, Czechoslovakia.

**Third European Conference on Clinical Oncology and Cancer Nursing**

*16–20 June 1985 Stockholm, Sweden*

ECCO 3, Box 1617, S-111 86 Stockholm, Sweden.

**International Symposium on Hexachlorobenzene (HCB)**

*24–28 June 1985 Lyon, France*

Dr J.R.P. Cabral, IARC, 150 cours Albert Thomas, 69372 Lyon Cedex 08, France. Tel. (7)875-81-81. Telex 380023 F

**Twelfth International Symposium for Comparative Research on Leukemia and Related Diseases**

*7–12 July 1985 Hamburg, FRG*

Dr D.S. Yohn, Secretary-General, Suite 302, 410 West 12th Avenue, Columbus, Ohio 43210, USA.

**Fourth World Conference on Lung Cancer**

*25–30 August 1985 Toronto, Canada*

IV World Conference on Lung Cancer, Secretariat Office, 342 MacLaren Street, Ottawa, Ontario, Canada.

**European Nuclear Medicine Congress 1985**

*3–6 September 1985 London, UK*

European Nuclear Medicine Congress 1985, Institute of Nuclear Medicine, Middlesex Hospital Medical School, Mortimer Street, London W1N 8AA, UK.

**International Symposium on Ovarian Cancer**

*11–13 September 1985 Glasgow, UK*

Dr A. Belfield, Biochemistry Department, Royal Maternity Hospital, Glasgow G4 0NA, UK.

**Conference on Chemical Modifiers of Cancer Treatment**

*20–24 October 1985 Clearwater, Florida*

Ms Suzanne Bohn, American College of Radiology, 925 Chestnut Street, Philadelphia, PA 19107, USA.